# Design Analysis and Experimental Study of Robotic Chair for Proton Heavy Ion Radiotherapy

**DOI:** 10.1155/2019/6410941

**Published:** 2019-12-01

**Authors:** Yongde Zhang, Zhikang Yang, Jingang Jiang, Xuesong Dai, Peiwang Qin, Shijie Guo, Sihao Zuo

**Affiliations:** ^1^Intelligent Machine Institute, Harbin University of Science and Technology, Harbin 150080, China; ^2^Hebei University of Technology, Xiping Road Beichen District, 300401 Tianjin, China; ^3^Foshan Baikang Robot Technology Co., Ltd., Shishan Town, Nanhai District, 528237 Foshan, China

## Abstract

Proton heavy ion radiotherapy is widely used and currently represents the most advanced radiotherapy technology. However, at present, proton heavy ion radiotherapy chairs in fixed beam radiotherapy rooms do not have a head and neck positioning function. This paper presents a novel design for a proton heavy ion radiotherapy chair with a head and neck positioning device. The design of the posture adjustment mechanism and the head and neck positioning device of the treatment chair is based on U-TRIZ theory and ergonomics, respectively. A positive kinematic analysis of the posture adjusting mechanism was carried out, as well as a workspace analysis of the head and neck positioning device. Finally, positioning error experiment and ergonomic evaluation were performed on a prototype of the head and neck positioning device. The proposed design of the treatment chair satisfies the requirements for posture adjustment and achieves the head and neck positioning function. The experimental results also provide a basis for further optimization of the design.

## 1. Introduction

Proton heavy ion radiotherapy is a new type of radiotherapy that has stoked international interest in recent years and represents the most advanced radiotherapy technique and is the future trend for radiotherapy treatments for cancers [1]. The characteristics of the proton heavy ion beam include an inverse dose distribution and the formation of a sharp Bragg peak when entering the human body. The ion beam is highly lethal to tumors and accurately attacks tumor cells, which reduces damage to surrounding tissue. In addition, it induces double-strand DNA breaks, thereby reducing the chances of tumor recurrence and metastasis [2–5].

During proton heavy ion radiotherapy treatments, the sitting posture of the patients with head and neck cancer, lung cancer, orthopnea [6], dyspnea, and dysphagia is necessary. To meet the needs of the sitting posture for radiotherapy, a number of scientific research institutes and medical device companies around the world have introduced special treatment chairs.

Schardt and Heeg designed a posture adjustment chair for patients with head and neck cancer in the heavy ion beam treatment room [7]. In 2006, Sommer et al. proposed a series of positioning chairs for radiotherapy patients [8]. The Orsay Proton Therapy Center in France developed a robotic chair for the treatment of head, neck, and eye tumors. Similarly, Chengdu Dr. Technology Co., Ltd. invented an automatic positioning chair for proton and heavy ion treatment of head and neck cancer [9]. In 2017, Guangdong Hengju Medical Technology Co., Ltd. designed a sitting posture fixation device for particle radiotherapy [10].

Nonetheless, existing radiotherapy chairs are still unsuitable for proton heavy ion fixed beam radiotherapy rooms and do not completely meet the requirement for six-degree-of-freedom (6-DOF) posture adjustment. Moreover, current thermoplastic film head and neck fixation technologies are time-consuming, uncomfortable for the patient, and not universally applicable.

This paper presents the design of a proton heavy ion radiotherapy chair with a head and neck positioning function. The design was applied to the posture adjustment and head and neck positioning of patients in a fixed beam radiotherapy room. The device facilitates the application of the fixed beam of proton heavy ion irradiation of tumors from different directions and angles, enhances the effects of radiotherapy, and improves the efficiency, versatility, and comfort of head and neck positioning.

## 2. Methods

### 2.1. Mechanical Structure Design of Treatment Chair

#### 2.1.1. Functional Requirement Analysis of Treatment Chair

**Figure 1:**
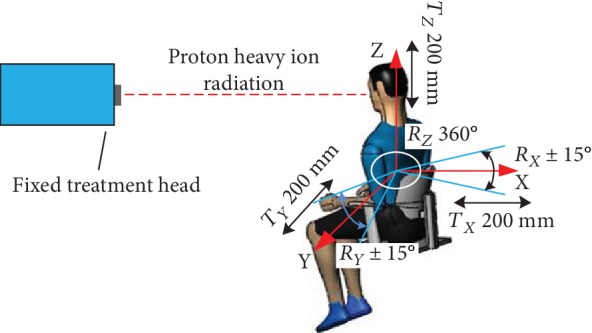
The function demand analysis of the posture adjustment.

For proton heavy ion radiation therapy, a more complex and larger treatment system is needed than the system required for conventional X-ray and electron linear accelerators. The working principle of the proposed system is illustrated in Table 1 [11]. The treatment chair for sitting position radiotherapy with a proton heavy ion fixed beam must provide two functions: posture adjustment and head and neck positioning. The posture adjustment function enables the irradiation of tumors from different directions and angles using the fixed beam. Specific adjustment parameters include 200 mm movements in the X, Y, and Z directions, ±15° rotations about the *x*-axis and *y*-axis, and 360° rotations about the *z*-axis, as shown in Figure 1. The head and neck positioning function must quickly and comfortably restrict the degrees of freedom of the head and neck depending on the somatotype of the patient.

#### 2.1.2. Design of Posture Adjustment Mechanism Based on U-TRIZ Theory

**Figure 2:**
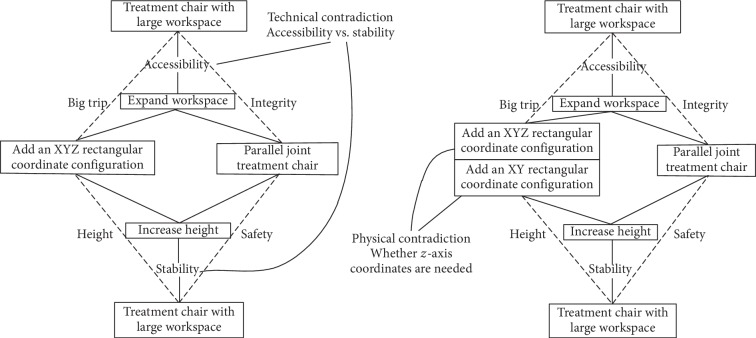
Technical contradiction and physical contradiction of treatment chair represented by the SAFC model.

U-TRIZ theory is a function-oriented and attribute-centered TRIZ theory of inventive problem solving, which unifies several tools and methods, and is an effective tool for innovative design [12, 13]. The 6-DOF parallel platform has a small workspace and cannot meet the needs of patients that must move 200 mm in the X, Y, and Z directions. Thus, it is necessary to add an XYZ rectangular coordinate slipway to the parallel platform. However, this increases the height in the Z direction, reduces stability, and creates redundant degrees of freedom. Therefore, the SAFC model of U-TRIZ theory was used for the present analysis, as shown in Figure 2.

**Figure 3:**
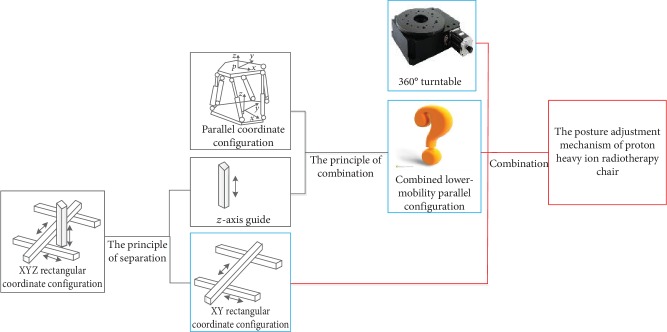
The analysis of posture adjustment mechanism based on U-TRIZ theory.

The technical contradiction and corresponding physical contradiction of the treatment chair design are shown in Figure 2. For the physical contradiction, the *z*-axis is separated from the XYZ rectangular coordinate configuration, then the *z*-axis is incorporated into the parallel configuration using the 40 inventive principles to produce a combined lower-mobility parallel coordinate configuration, which can only be adjusted by modifying the X and Y angles. Finally, the XY plane coordinate configuration, lower-mobility parallel configuration, and 360° turntable are combined to create the posture adjustment mechanism of the proton heavy ion radiotherapy chair, as shown in Figure 3.

**Figure 4:**
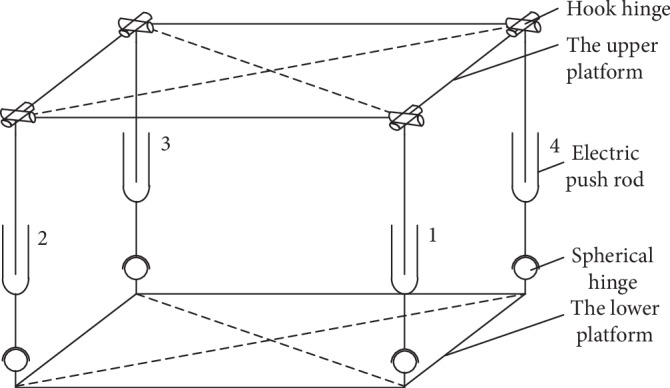
The common parallel mechanism.

**Figure 5:**
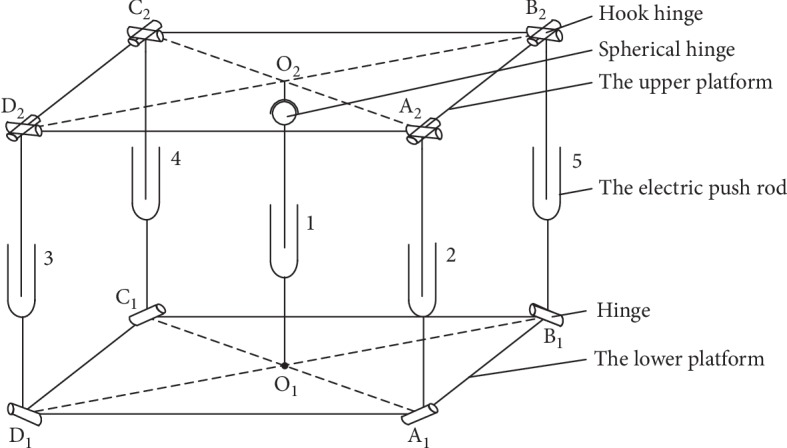
The schematic diagram of combined lower-mobility parallel mechanism.

The above scheme adds a bottom-fixed vertical lift electric push rod along the Z direction of the ordinary parallel mechanism, as shown in Figure 4, and replaces the spherical hinges at the lower end of the four branches with a single hinge by grouping them into a lower-mobility parallel mechanism, as shown in Figure 5. Electric push rod 1 is represented by O_1_O_2_ along the vertical direction of the *z*-axis. The bottom of the rod is fixed to the lower platform and the top is connected to the upper platform by spherical hinges, which allow vertical movements. Two active adjustment branches, A_1_A_2_ and B_1_B_2_, are connected to the lower platform via two hinges at A_1_ and B_1_ that rotate around the pin. Electric push rods 2 and 3 are located in the middle and connected to the upper platform via hook hinges at A_2_ and B_2_. Two passive adjustment branches, C_1_C_2_ and D_1_D_2_, are connected in the same way as A_1_A_2_ and B_1_B_2_ and follow the stretching motion of the A_1_A_2_ and B_1_B_2_ branches while playing a supporting role. The degrees of freedom of the combined lower-mobility parallel mechanism can be determined as follows:

(1) $$\begin{array}{c} M_{1}=6\left(n-g-1\right)+\overset{n}{\underset{i=1}{\sum }}f_{i}=6\times \left(12-14-1\right)+20=2. \end{array}$$

The DX3535A XY mobile unit (THK, Japan) and GT-135C single-stage high-precision hollow rotary platform reducer (Liming, Taiwan) were selected for generating translational motion in the X and Y directions and rotary motion in the Z direction, respectively.

**Figure 6:**
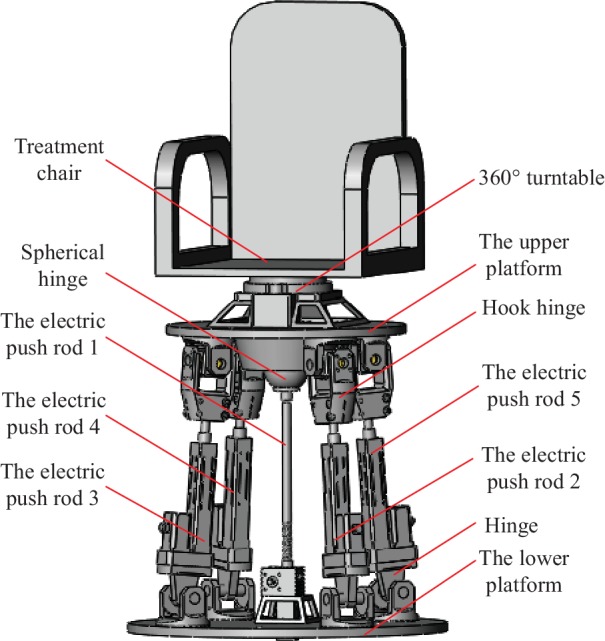
The 3D model of the proton heavy ion radiotherapy chair posture adjustment mechanism.

For the analysis, a three-dimensional (3D) model of the proton heavy ion radiotherapy chair posture adjustment mechanism was established and is presented in Figure 6. The working principles of the mechanism can be described as follows: the XY moving unit and electric push rod 1 are adjusted to place the patient in a predetermined position, then the length of the electric push rods 3 and 5 is adjusted to rotate the patient a certain angle about the *x*-axis, the length of electric push rods 2 and 4 is adjusted to rotate the patient a certain angle about the *y*-axis, and finally, the 360° turntable is adjusted to rotate the patient a certain angle about the *z*-axis.  Table 2 lists the performance parameters of the posture adjustment mechanism.

#### 2.1.3. Design of Universal Head and Neck Positioning Device Based on Ergonomics

During the medical device design process, ergonomic standards typically include the size, appearance, convenient operation, and safety of the product [14, 15]. This paper adopts the ergonomic standards to modularize the universal head and neck positioning device. The three main submodules are the head circumference positioning, chin positioning, and backboard.

*(1) Head Circumference Positioning Module*. The function of the head circumference positioning module is to position the head from the plane of the head circumference and the top of the head, by setting three positioning points at equal angles on the head circumference of approximately elliptical shape and an anchor point on the top of the head. To realize the final position, the DOF of the head is restrained.

**Figure 7:**
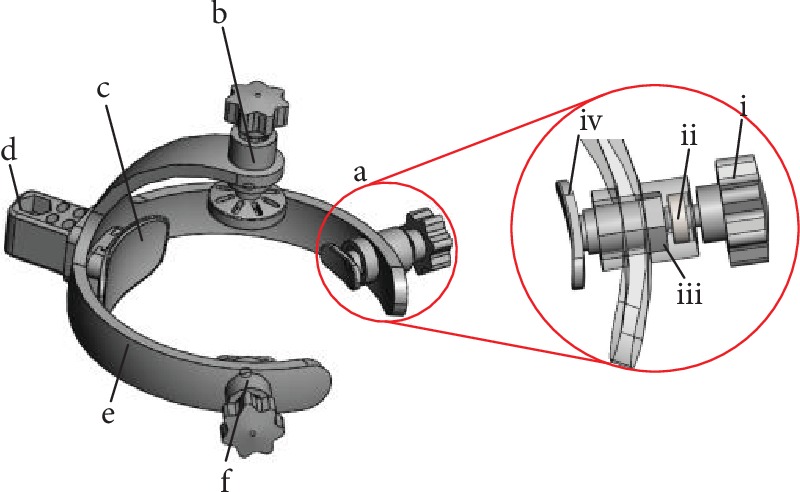
The 3D structure of the head circumference positioning module. (a) The adjustable head circumference positioning block 1 with (i) the hand-operated handle screw, (ii) bearing, (iii) screw nut, and (iv) the positioning block. (b) The adjustable head top positioning block. (c) The fixed head circumference positioning block. (d) The height adjustment mechanism. (e) Head circumference positioning bracket frame. (f) The adjustable head circumference positioning block 2.

A 3D model of the head circumference positioning module was established, as shown in Figure 7. The working principle can be described as follows: a hand-operated handle screw passes through the special bearing end cover to match the inner diameter of the bearing; the outer diameter of the bearing is fixed to match the circular hole on the curved bracket of the positioning block and can be rotated; to fix the bearing, a special bearing end cover is inserted into the circular hole on the curved bracket of the positioning block; and the screw nut matches the hand-operated handle screw and can move back and forth along the hexagonal prism hole in the head positioning block to realize front and rear adjustments of the positioning block. The same adjustment method was adopted for the adjustable head circumference positioning blocks 1 and 2 as well as the adjustable head circumference positioning block.

The size of the human body determines the geometric space and range of motion required for the design and is the basic information used in the man-machine system or product designs. In the present design, the size parameter range of the head and neck of the human body must be considered so that the final product meets the needs of different body types and improves patient comfort.

**Figure 8:**
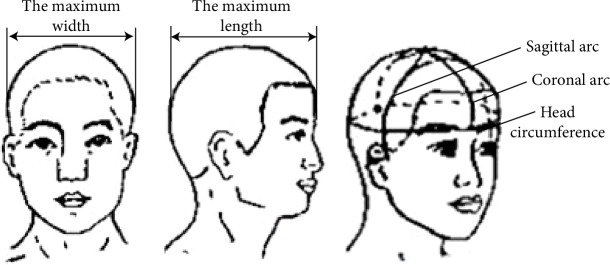
Human head parameters.

Head- and neck-related parameters were obtained from the Chinese standard GB10000-88 “Human dimensions of Chinese adults,” as shown in Figure 8, which provided basic values for the size of Chinese adults according to ergonomic requirements. The standard is applied to technical product upgrades and labor safety protection for industrial products, architectural design, military applications, and in other industries. Based on this standard, the inner diameter of the ring where the positioning block is placed was set to 120 mm, the external diameter was set to 130 mm, and the wall thickness was 10 mm.

*(2) Chin Positioning Module*. The function of the chin positioning module is to fully position the head using the head circumference positioning module. According to ergonomic standards, the shape of the chin rest must be as close as possible to the actual shape of the chin and can be adjusted to enhance the position of the chin.

**Figure 9:**
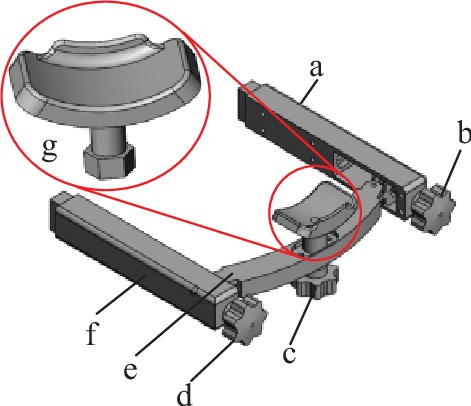
Chin positioning adjustment module. (a) The support arm 1. (b) The hand-operated handle screw 1. (c) The hand-operated handle screw 2. (d) The hand-operated handle screw 3. (e) Cantilever. (f) The support arm 2. (g) The chin block.

A 3D model of the chin positioning module was established and is shown in Figure 9. The working principle can be described as follows: a screw-nut slider is installed within long slots in support arms 1 and 2; a cantilever is mounted on the left and right screw-nut sliders, and the front and rear positions are adjusted by the hand screw handles 1 and 2; the chin rest is installed in the middle of the cantilever, and to move the chin support up and down, the screw nut is adjusted using hand screw handle 3.

Based on the configuration analysis, the chin positioning block can only be used for front and rear and up and down adjustments; therefore, it is only necessary to calculate the up and down and front and rear adjustment lengths. Consulting GB10000-88, the maximum length of the head is 161~200 mm, the length of the back of the measuring head to the back of the plate is 35 mm, such that the chin block before and after back position adjustment is 196~235 mm.

*(3) Backboard*. The backboard supports the installation of the head circumference positioning module and the chin rest positioning module and must meet the installation, positioning, and adjustment requirements of the two modules. Consulting GB10000-88, the height range from the shoulders to the top of the head is 283~446 mm and the shoulder width range is 304~417 mm. The height of the backboard must be adjusted according to the height of the patient while in a seated position, and the lower edge is always the same height as the shoulders of the human body to position the head. A backboard size of 500 × 600 mm was selected.

**Figure 10:**
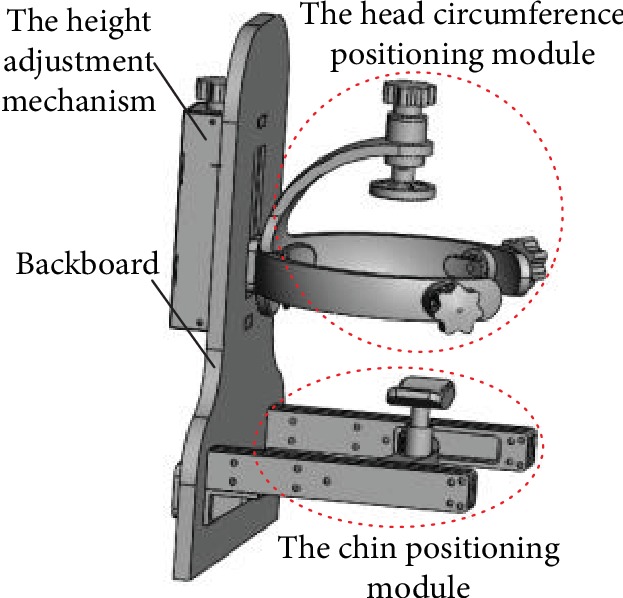
The head and neck positioning device of proton heavy ion radiotherapy chair.

The head circumference positioning module, chin positioning module, and backboard were assembled, and the 3D structure of the proton heavy ion radiotherapy chair head and neck positioning device was determined, as shown in Figure 10.

### 2.2. Analysis of Kinematic Performance of Treatment Chair

#### 2.2.1. Positive Kinematic Analysis of Posture Adjustment Mechanism

**Figure 11:**
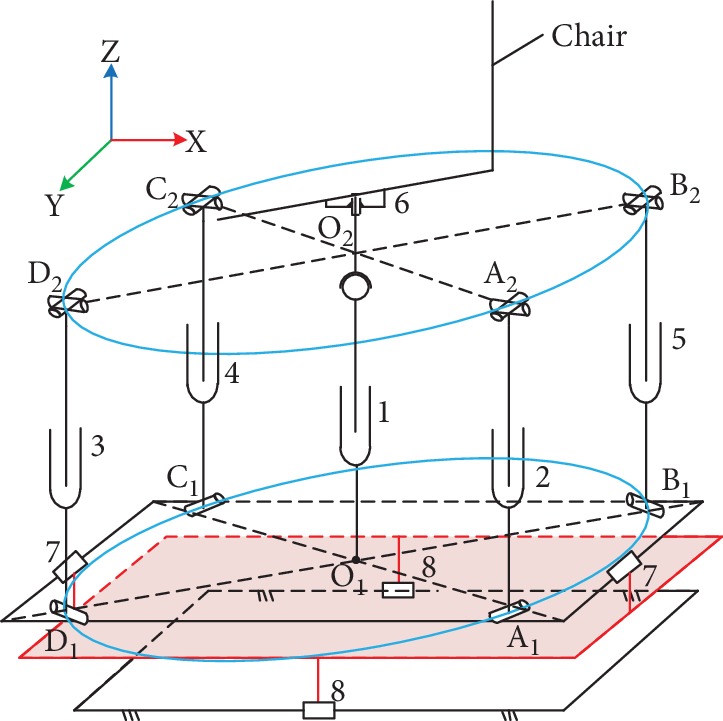
Structural diagram of the chair posture adjustment mechanism.

A schematic drawing of the 3D model of the proposed posture adjustment mechanism is presented in Figure 11. Posture adjustment can be divided into two parts: position adjustment and angle adjustment. In the whole posture adjustment mechanism, movement of the lower-mobility parallel mechanism is more complicated with X and Y angle adjustment functions. For this reason, a kinematic analysis was only performed on the X and Y angle adjustment parallel platforms.

**Figure 12:**
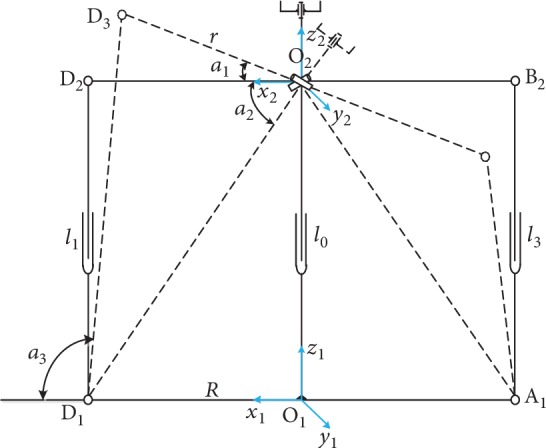
*x*-axis angle adjustment structure diagram.

As shown in Figure 11, the X and Y angle adjustment parallel platform has four branches including two pairs of symmetrical branches, one pair for the *x*-axis angle adjustment and the other pair for the *y*-axis angle adjustment. The *x*-axis angle adjustment was separately analyzed first. The structure is shown in Figure 12.

The base coordinate system O_1_‐*x*_1_*y*_1_*z*_1_ was established on the bottom plane, and the upper moving plane was used to establish the moving coordinate system O_2_‐*x*_2_*y*_2_*z*_2_; *R* is the length from the center of the bottom platform to the electric push rod; *r* is the length from the center of the upper moving platform to the electric push rod, *R* = *r* = O_1_A_1_ = O_1_B_1_ = O_1_C_1_ = O_1_D_1_ = O_2_A_2_ = O_2_B_2_ = O_2_C_2_ = O_2_D_2_ = 270 mm; *l*_0_ is the length of the lifting electric push rod, its values range from 360 to 600 mm; *l*_1_ and *l*_3_ are the length of the *x*-axis angle adjustment electric push rod with values ranging from 360 mm to 720 mm.

In Figure 12, *α*_1_ is the rotation angle of the upper moving platform. The loop closure equation can be established using the vector method.

(2) $$\begin{array}{c} \bar{{\text{O}}_{1}{\text{D}}_{1}}+{\bar{{\text{D}}_{1}\text{D}}}_{2}={\bar{{\text{O}}_{1}\text{O}}}_{2}+{\bar{{\text{O}}_{2}\text{D}}}_{2}. \end{array}$$

From equation (2),

(3) $$\begin{array}{c} \left.\begin{array}{c} x_{1}‐\text{axis component}:R+l_{1}\cos\alpha_{3}=0+r\cos\alpha_{1}\\ z_{1}‐\text{axis component}:0+l_{1}\sin\alpha_{3}=l_{0}+r\sin\alpha_{1} \end{array}\right\}. \end{array}$$

In ΔD_1_D_3_O_2_, according to the cosine theorem,

(4) $$\begin{array}{c} \cos\left(\alpha_{1}+\alpha_{2}\right)=\left(\frac{r^{2}+l_{0}^{2}+R^{2}-l_{1}^{2}}{2r\sqrt{R^{2}+l_{0}^{2}}}\right), \end{array}$$

where *α*_2_ = ∠D_1_O_2_D_2_, and the value changes as the lift electric push rod O_1_O_2_ elongates.

(5) $$\begin{array}{c} \alpha_{2}=\arctan\frac{l_{0}}{R}. \end{array}$$

Therefore, the value of *α*_2_ ranges from 53.13° to 65.77°.

Then, the relationship between the *x*-axis adjustment angle *α*_1_ and the length of the branch can be derived from equation (4) as

(6) $$\begin{array}{c} \alpha_{1}=\arcsin\left(\frac{r^{2}+l_{0}^{2}+R^{2}-l_{1}^{2}}{2r\sqrt{R^{2}+l_{0}^{2}}}\right)-\alpha_{2}=\arcsin\left(\frac{r^{2}+l_{0}^{2}+R^{2}-l_{1}^{2}}{2r\sqrt{R^{2}+l_{0}^{2}}}\right)-\arctan\frac{l_{0}}{R}, \end{array}$$

where *R* is the length from the center of the platform to the electric push rod, *r* is the length from the center of the upper moving platform to the electric push rod, *l*_0_ is the length of the lifting electric push rod, and *l*_1_ is the length of the *x*-axis angle adjusting the electric push rod.

Similarly, the relationship between the *y*-axis adjustment angle *β*_1_ and the length of its branch is

(7) $$\begin{array}{c} \beta_{1}=\arcsin\left(\frac{r^{2}+l_{0}^{2}+R^{2}-l_{2}^{2}}{2r\sqrt{R^{2}+l_{0}^{2}}}\right)-\beta_{2}=\arcsin\left(\frac{r^{2}+l_{0}^{2}+R^{2}-l_{2}^{2}}{2r\sqrt{R^{2}+l_{0}^{2}}}\right)-\arctan\frac{l_{0}}{R}, \end{array}$$

where *l*_2_ is the length of the *x*-axis angle adjustment electric push rod.

**Figure 13:**
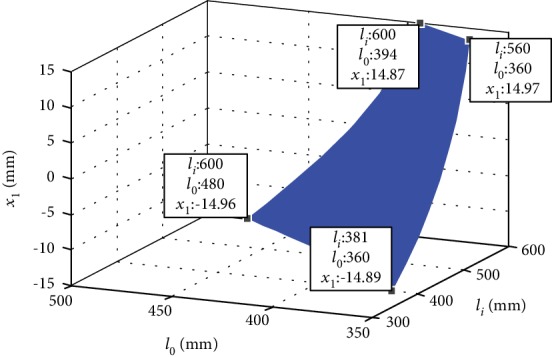
Electric push rod elongation and angle adjustment.

From equations (6) and (7), the relationship between the *x*-axis and *y*-axis adjustment angle *x*_1_ (*x* = *α*, *β*) and the length of the branch can be obtained. The effective adjustment range of the *x*-axis and *y*-axis adjustment angle is -15°~15°, the variable *x*_1_ (*x* = *α*, *β*) ranges from -15° to 15°. The relationship between the three *x*-axis variables and *y*-axis adjustment angle *x*_1_ (*x* = *α*, *β*), electric push rod elongation *l*_0_, and electric push rod elongation *l*_*i*_ (*i* = 1, 2) is shown in Figure 13.

The four extreme position coordinates indicate that the *x*-axis and *y*-axis adjustment angle *x*_1_ (*x* = *α*, *β*) is between -15° and 15°, the length of the electric push rod *l*_0_ is adjustable from 360 mm to 480 mm, the length of the electric push rod *l*_*i*_ (*i* = 1, 2) is adjustable from 381 mm to 480 mm, and the length of the electric push rods *l*_3_ and *l*_4_ can also be adjusted according to the elongation or shortening of *l*_1_ and *l*_2_.

#### 2.2.2. Workspace Analysis of Head and Neck Positioning Device

In robotics, the workspace or reachable workspace is defined as the set of all the target points that can be reached by the end effector when different joints of the robot are in motion. The work ability of the robot is an important kinematic index [16, 17].

For the proton heavy ion radiotherapy process, whether the universal head and neck positioning device of the treatment chair workspace can meet the head and neck positioning requirements of different body types is important and is the basis for measuring the positioning function of the head and neck.

In the present study, the second-generation MATLAB/SimMechanics library was used for the modeling analysis, and the position of the head top positioning block, head circumference positioning block, fixed head circumference positioning block, and chin positioning block was recorded in the base coordinate system.

**Figure 14:**
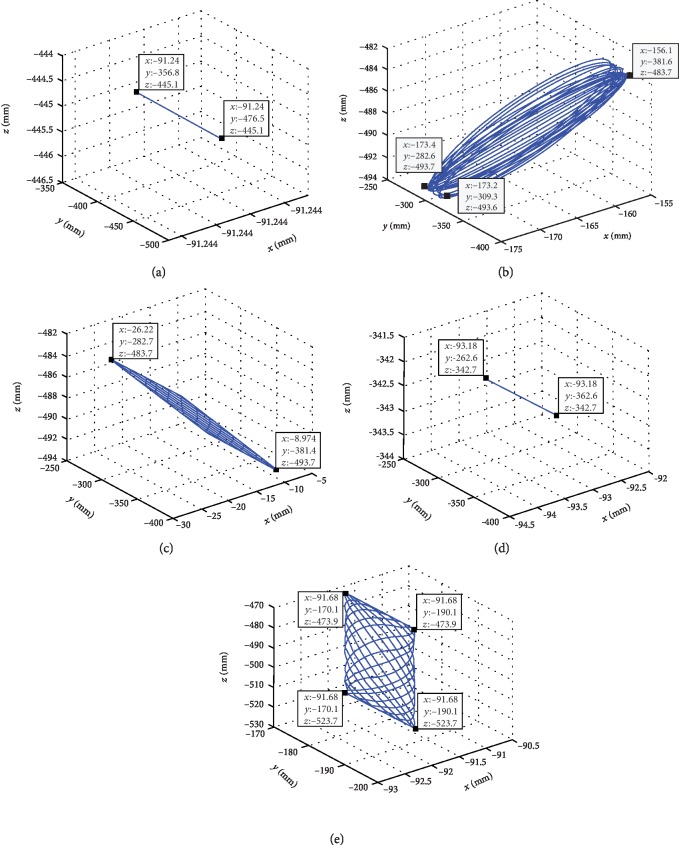
Angle adjustment relationship between *x*-axis and *y*-axis. (a) The workspace of head top positioning block. (b) The workspace of head circumference positioning block 1. (c) The workspace of head circumference positioning block 2. (d) The workspace of fixed head circumference positioning block. (e) The workspace of chin positioning block.

The simulated workspace of each positioning submodule of the head and neck positioning device is illustrated in Figure 14. The workspace simulation of the above positioning module shows that each module can meet the head and neck positioning requirements of patients.

## 3. Results and Discussion

**Figure 15:**
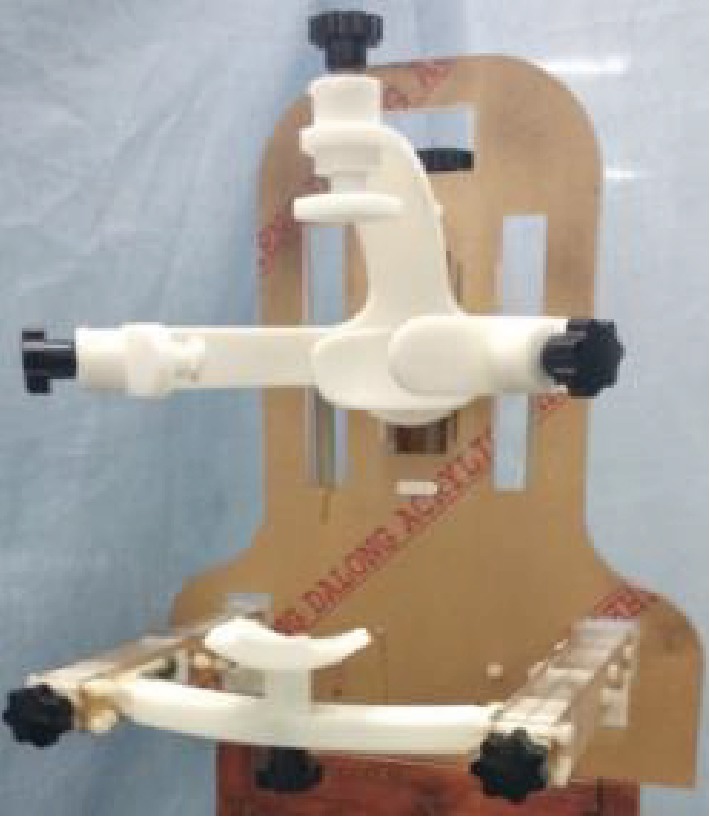
The universal prototype of the head and neck positioning.

The head and neck positioning device is made of PMMA and plastic to avoid radiation damage to the patient and operators. A prototype of the head and neck positioning device shown in Figure 15 was assembled using an acrylic plate and resin material.

### 3.1. Positioning Error Test of Each Module of Positioning Device

As shown in Table 3, the chin positioning block has four adjustment limits: proximal low point, proximal high point, distal low point, and distal high point. The center position of the bottom of the backboard was taken as the coordinate origin (0, 0, 0), and a rectangular spatial coordinate system was established. The positive Z direction was set as vertically upwards, the positive direction of the *y*-axis was set as the outward-facing direction of the vertical backboard, and the *x*-axis coincides with the lower edge of the backboard and the positive X direction was set to the right. The spatial coordinate position of the center of the concave chin block was measured and compared with the corresponding data calculated during the design stage.

It can be seen from Table 3 that the maximum deviation of the *y*-axis is 2.5 mm in the process of assembling and adjusting the chin positioning block, which differs quite considerably from results of the finite element analysis. There are two reasons such as large deviation: errors in processing and assembling the parts and the two lead screws are not synchronized when the chin block is adjusted. Owing to this, the chin positioning block cannot be fully adjusted at the distal and proximal ends; however, the maximum deviation along the *x*-axis and *z*-axis was still less than 2.5 mm. This meets the positioning requirements, and the chin positioning module of the prototype can be further optimized using the same analysis.

**Figure 16:**
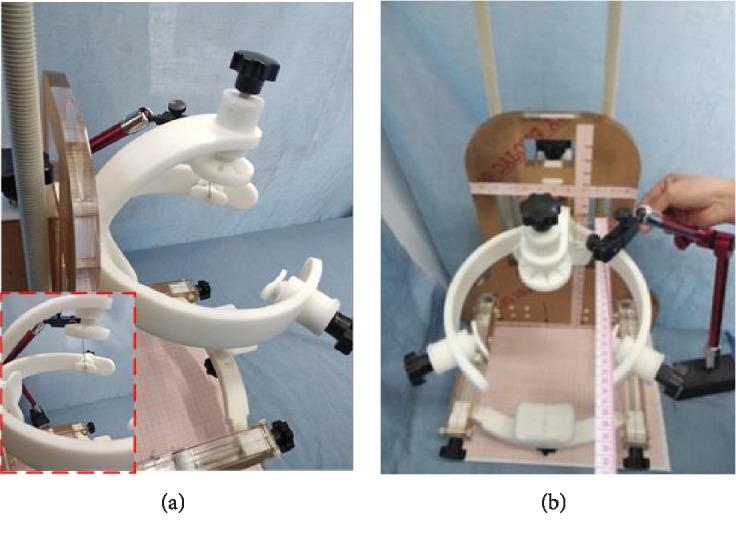
The universal prototype of the head and neck positioning. (a) The equipotential point mark of head circumference positioning block. (b) The equipotential point measurement.

The equipotential method was adopted to measure the block parameters for the head circumference positioning using adjustable head circumference positioning block. First, a point at the end of the head circumference positioning block was marked and the head circumference was adjusted to the position limit; the flexible manipulator was adjusted so that the end coincides with the end of the head circumference positioning bracket; then, the end point center of the head circumference positioning block was replaced with the end of the manipulator, which is easier to measure, as shown in Figure 16(a). The head circumference positioning block was adjusted to other positions, and the end point of the manipulator is measured, as shown in Figure 16(b). The manipulator data includes the position of the center point of the head circumference positioning block. The head top positioning parameter was also measured using this method.

Based on the above method, coordinates and errors of the end center position of the head circumference positioning block and the head top positioning block are shown in Table 4.

From Table 4, the error of the head circumference positioning block along the *y*-axis and *z*-axis is large, and the error along the *z*-axis is a maximum of 3 mm. The head top positioning block also exhibited large errors along the *y*-axis and *z*-axis, and the maximum error was 2 mm.

When the head of the patient is positioned, the top of the head generates an upward supporting force that acts on the head circumference positioning bracket frame and reduces the error. The experimental results provide reference values for further design improvements and balance the error caused by the self-weight of the head circumference positioning bracket frame.

### 3.2. Ergonomic Evaluation Experiment

**Figure 17:**
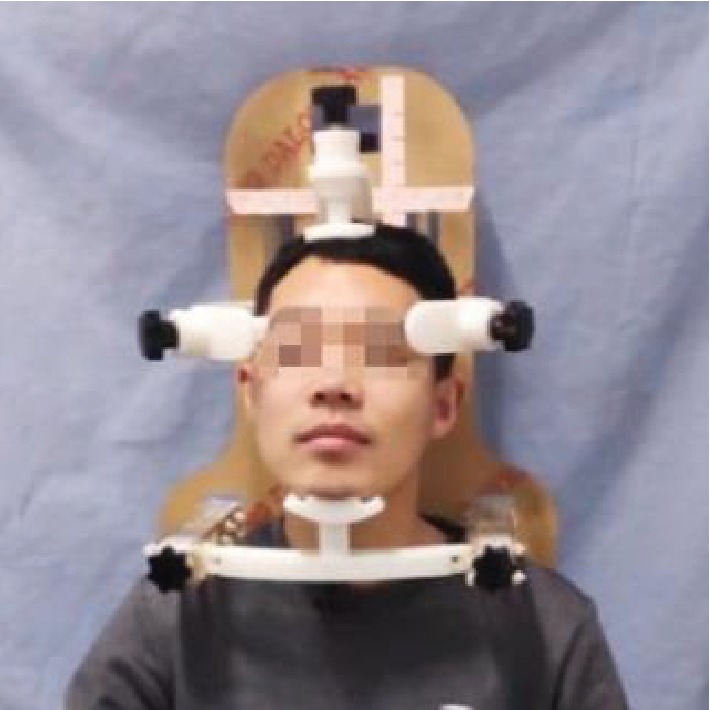
The experiment of head and neck positioning.

**Figure 18:**
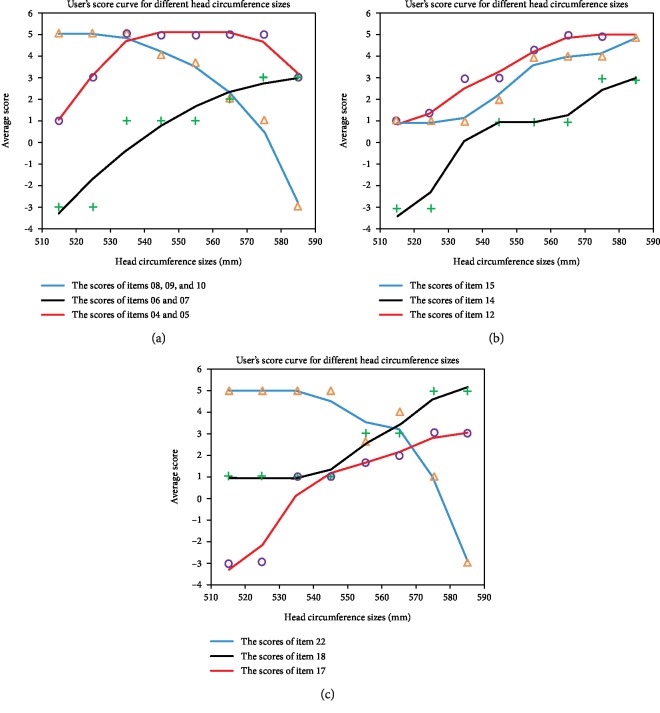
The evaluation scoring curve of universal head and neck positioning device. (a) The evaluation scoring curve of head circumference positioning module. (b) The evaluation scoring curve of chin positioning module. (c) The evaluation scoring curve of backboard module.

The universal head and neck positioning device was designed from an ergonomic perspective; therefore, the design can be evaluated using human-machine system evaluation methods, which are generally divided into three categories: experimental methods, simulations, and actual operational measurements [18–21]. The present study selected the experimental approach to evaluate the general purpose of the head and neck positioning device. The following experimental procedure was carried out:

1. An evaluation form was prepared from the perspective of “human, machine, and environment” and evaluated and compiled using ergonomic principles and details of the universal head and neck positioning device, as shown in Table 5. Collected data included the size of a user's head and 22 additional measurements from the three submodules. The evaluation data was then divided into five grades according to user experience, thereby providing feedback on the rationality of the modular design
2. An evaluation sample was defined based on the selection criteria. The main factor affecting positioning in this experiment was the head circumference, which was selected according to the normal distribution. A total of 12 human volunteers were selected for the evaluation. The head size distribution is presented in Table 6
3. Head and neck positioning experiments were performed. Briefly, the head and neck of volunteers participating in the experiment were positioned, as shown in Figure 17, and the evaluation form was filled out based on their experiences
4. The evaluation results were statistically analyzed. Evaluation scores were counted for all items according to the head circumference, average scores were calculated, and evaluation score curves were plotted, as shown in Figure 18

From the curves in Figure 18(a), it can be observed that the expansion of the adjustable positioning block meets the needs of users with moderate head dimensions but can also meet the needs of larger and smaller users. Smaller users gave higher evaluation scores for the comfort of each positioning block, but the scores of larger users were poor.

Overall, the adjustment efficiency of the adjustable positioning block was low, and since the larger user is taller than the small user, only small length adjustments are needed for the large user. From Figure 18(b), it can be seen that the size of the chin positioning block is more suitable for larger users and small users gave slightly lower evaluation scores.

In general, adjustment efficiency scores of the chin positioning block were fairly low, with scores of large users only slightly higher because the length must only be adjusted small amount. Moreover, larger users found the device more comfortable than small users. It can be seen from Figure 18(c) that the positioning block mounting efficiency is higher for large head circumferences. The backplane was more difficult for smaller users to adjust, whereas the size of the head space was more suitable for small- and medium-sized users; therefore, comfort for large users was poor.

In summary, the universal head and neck positioning device can better meet the needs of users of medium head sizes and must be further optimized to better meet the needs of small and large users.

## 4. Conclusions

This paper presented the design of a proton heavy ion radiotherapy chair with a head and neck localization function. The design of the posture adjustment module and head and neck positioning module was based on U-TRIZ theory and ergonomics, respectively. A positive kinematic analysis of the posture adjustment mechanism of the treatment chair and workspace analysis of the head and neck positioning device were carried out, as well as a stiffness and strength analysis under several limit states. Finally, a positioning error experiment and ergonomics evaluation were performed using a prototype of the universal head and neck positioning device. The results show that the head and neck positioning device can meet the head and neck positioning requirements of medium-sized users much better than existing chairs; however, further optimization is still needed to meet the needs of all users.

## Figures and Tables

**Table 1:** The proton heavy ion radiotherapy work process.

Order number	Working principle decomposition diagram	Principle description
a	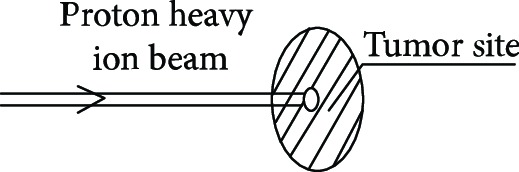	The proton heavy ion beam is accelerated by the accelerator and becomes a high-energy proton heavy ion beam.

b	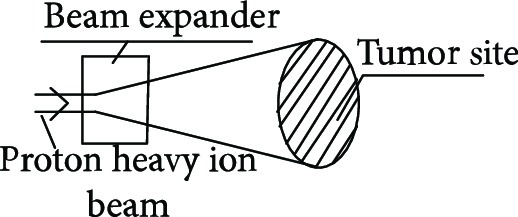	High-energy proton heavy ion beams need to be expanded to cover the entire tumor site.

c	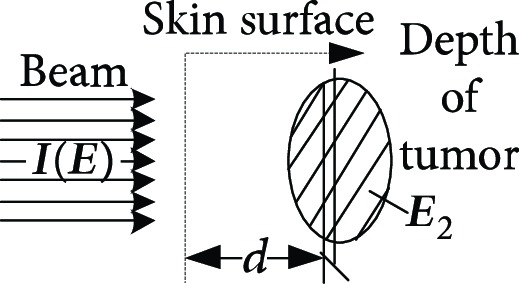	Select proton heavy ion beams of different energies for tumors of different depths.

d	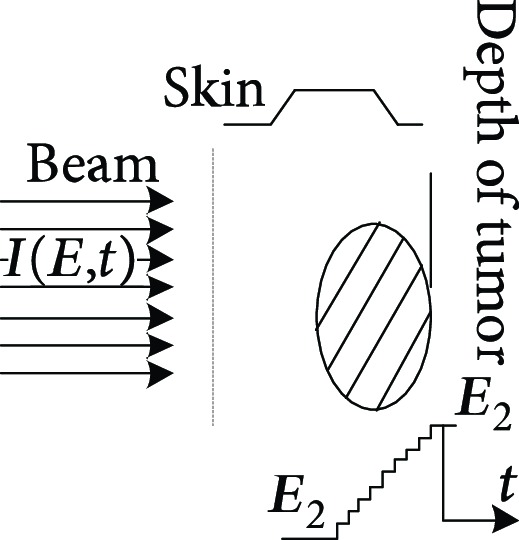	The energy modulator is used to modulate the energy of the proton heavy ion beam, gradually increasing its energy and achieving fine treatment.

e	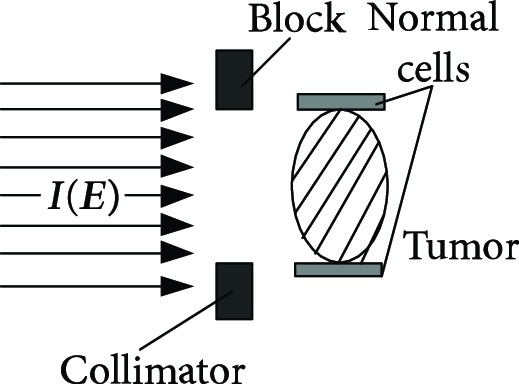	The beam collimator blocks the proton heavy ion beam directed to normal cells and avoids damage to normal cells.

f	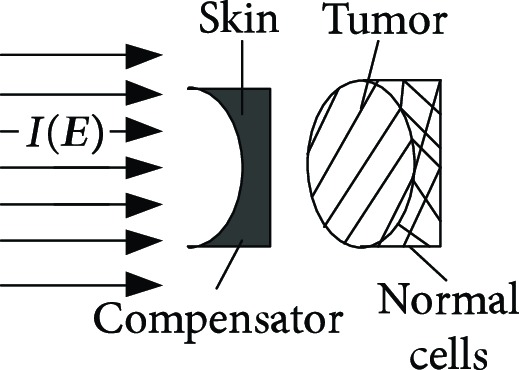	The proton heavy ion beam needs to pass through a special compensator at the front of the skin to prevent damage to normal cells at the posterior edge of the tumor.

g	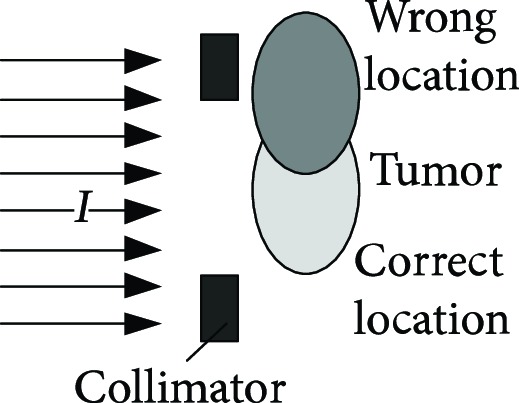	Introduce a precision positioning system to align the patient to the beam and avoid error irradiation.

h	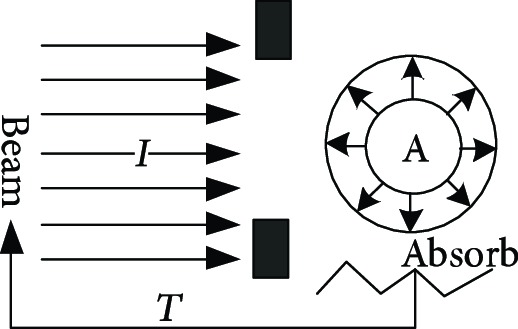	In order to synchronize the proton heavy ion beam irradiation with the breathing and avoid the influence of breathing on the tumor size, it is necessary to equip a respiratory door control system.

i	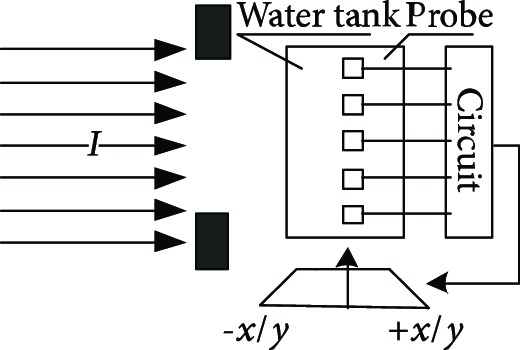	A measurement and calibration system is required to ensure uniformity of the proton dose in the longitudinal and transverse directions.

j	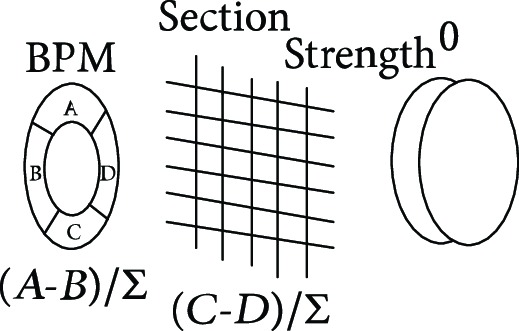	A set of beam monitoring system is introduced to monitor whether the beam center, strength, and section distribution meet the requirements in real time.

k	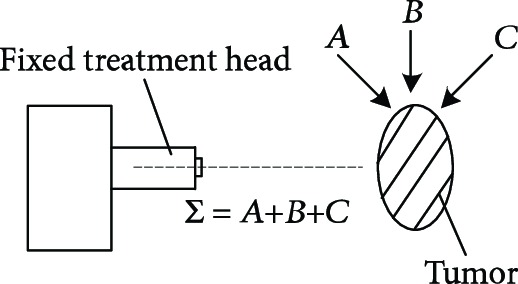	The end of the proton heavy ion beam output needs to have a posture adjustment mechanism, and the treatment head is irradiated from different angles in order to obtain a good efficacy.

**Table 2:** The performance parameters of treatment chair posture adjustment mechanism.

The effective travel in the *x*-axis (mm)	350
The effective travel in the *y*-axis (mm)	350
The effective travel in the *z*-axis (mm)	200
*x*-axis angle adjustment (°)	±20
*y*-axis angle adjustment (°)	±20
*z*-axis angle adjustment (°)	360
The maximum load (N)	2000
The number of DOF	6

**Table 3:** The adjustment parameter of chin positioning block.

Position		Proximal low point	Proximal high point	Distal low point	Distal high point
Calculating coordinates		(0, 165.0, 145.0)	(0, 165.0, 162.0)	(0, 235.0, 145.0)	(0, 235.0, 162.0)

Location description		Chin positioning block at the proximal low point	Chin positioning block at the proximal high point	Chin positioning block at the distal low point	Chin positioning block at the distal high point

Measurement diagram		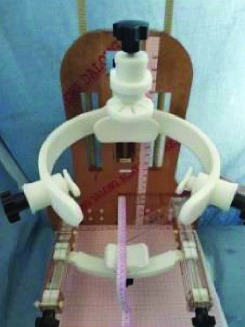	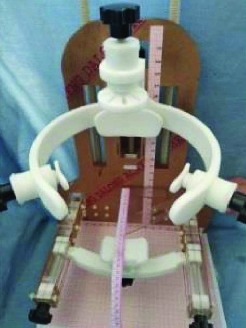	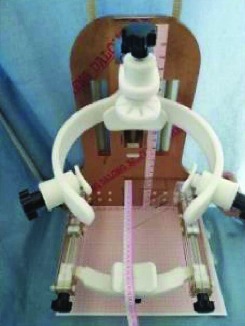	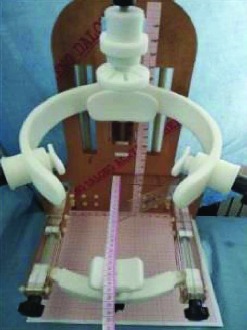

Measuring coordinates		(1.5, 167.0, 143.5)	(-1.5, 167.5, 161.0)	(1.0, 234.5, 143.0)	(0.5, 235.0, 160.5)

Error (mm)	Δ*x*	1.5	-1.5	1.0	0.5
	Δ*y*	2.0	2.5	-0.5	0
	Δ*z*	1.5	-1.0	-2.0	-1.5

**Table 4:** The coordinates and errors of the head circumference positioning block and the head top positioning block.

Module	State	Calculating coordinates	Measuring coordinates	Error (mm)		
				Δ*x*	Δ*y*	Δ*z*
Head circumference positioning block 1	High position and extension	(-64.7, 173.8, 360.0)	(-63.5, 174.5, 357.0)	1.2	0.7	-3.0
	High position and shorten	(-82.0, 183.8, 360.0)	(-84.0, 182.0, 358.0)	-2.0	-1.8	-2
	Low position and extension	(-64.7, 173.8, 260.0)	(-64.0, 173.5, 258.5)	0.7	-0.3	-1.5
	Low position and shorten	(-82.0, 183.8, 260.0)	(-81.0, 183.0, 261.0)	1.0	-0.8	1.0

Head circumference positioning block 2	High position and extension	(64.7, 173.8, 360.0)	(65.0, 171.0, 358.0)	0.3	-2.8	-2.0
	High position and shorten	(82.0, 183.8, 360.0)	(83.5, 183.0, 358.0)	1.5	-0.8	-2.0
	Low position and extension	(64.7, 173.8, 260.0)	(65.5, 174.5, 260.0)	0.8	0.7	0
	Low position and shorten	(82.0, 183.8, 260.0)	(80.0, 182.5, 259.0)	-2.0	-1.3	-1.0

Fixed head circumference positioning block 3	High position	(0, 34.5, 360.0)	(0, 35.0, 358.5)	0	0.5	-1.5
	Low position	(0, 34.5, 260.0)	(0, 33.5, 261.0)	0	-1.0	1.0

Head top positioning block	High position and extension	(0, 135.0, 430.0)	(1.0, 133.5, 428.5)	1.0	-1.5	-1.5
	High position and shorten	(0, 135.0, 450.0)	(0.5, 133.5, 449.0)	0.5	-1.5	-1.0
	Low position and extension	(0, 135.0, 330.0)	(0, 134.0, 328.0)	0	-1.0	-2.0
	Low position and shorten	(0, 135.0, 350.0)	(-0.5, 133.5, 348.5)	-0.5	-1.5	-1.5

**Table 5:** The evaluation form.

The evaluation form of the head and neck positioning device						
Through the subjective feelings of the volunteers during the experiment, the performance of the head circumference positioning module, the chin positioning module, and the backboard module was evaluated from the perspective of ergonomics.						

Name		Scoring standard: very suitable: 5, suitable: 3, general: 1, inappropriate: -3, very inappropriate: -5				
Gender						

Head size parameter	Head circumference	Head full height	Eye height	Maximum width of the head	Maximum length of the head	
Unit (mm)						

The universal head and neck positioning device evaluation	Submodule	Evaluation project			Score	Comment
	The head circumference positioning module evaluation	(01) Whether the adjustable head circumference positioning block 1 and 2 is suitable				
		(02) Whether the fixed head circumference positioning block 3 is suitable				
		(03) Whether the adjustable head top positioning block 4 is suitable				
		(04) Whether the expansion amount of adjustable head circumference positioning blocks 1 and 2 is suitable				
		(05) Whether the expansion amount of adjustable head top positioning block 4 is suitable				
		(06) Whether the adjustment efficiency of head circumference adjustable positioning blocks 1 and 2 is efficient				
		(07) Whether the adjustment efficiency of head top positioning block 4 is efficient				
		(08) Whether the adjustable head circumference positioning block 1, 2 is comfortable				
		(09) Whether the fixed head circumference positioning block 3 is comfortable				
		(10) Whether the adjustable head top positioning block 4 is comfortable				
		(11) Whether the head circumference positioning is stable				
	The chin positioning module evaluation	(12) Whether the chin positioning block is suitable				
		(13) Whether the adjustment amount of the chin positioning block is sufficient				
		(14) Whether the adjustment efficiency of the chin positioning block is efficient				
		(15) Whether the chin positioning block is comfortable				
		(16) Whether the support arms 1 and 2 press the shoulder				
	The backboard module evaluation	(17) Whether the head circumference positioning block frame is efficient				
		(18) Whether the height of the backboard is easy to adjust				
		(19) Whether the height adjustment of the backboard is stable				
		(20) Is the device safe?				
		(21) Is it easy to grasp the hand-held handle?				
		(22) Is the head space the right size?				

Suggest:						

**Table 6:** Volunteer head circumference size distribution table.

Head circumference size	510-519	520-529	530-539	540-549	550-559	560-569	570-579	580-589
Number	1	1	1	2	3	2	1	1

## Data Availability

The data used to support the findings of this study are available from the corresponding author upon request.

## References

[1] Li J. (2015). The project management practice of Shanghai Proton Heavy Ion Hospital. *China Engineering Consulting*.

[2] Li S. F., Shankou M. Z., Wang Z. J. (2011). Research status of heavy ion radiation therapy for malignant tumors. *Chinese Journal of Clinicians (Electronic Edition)*.

[3] Mizutani S., Takada Y., Kohno R., Hotta K., Tansho R., Akimoto T. (2016). Application of dose kernel calculation using a simplified Monte Carlo method to treatment plan for scanned proton beams. *Journal of Applied Clinical Medical Physics*.

[4] Wang L., Dai X. Y. (2016). Current status and prospects of global proton heavy ion hospitals. *Chinese Hospital Architecture & Equipment*.

[5] Xie J. X., Zhang L. (2016). Proton/heavy ion radiotherapy technology and application. *China Medical Device Information*.

[6] National Taiwan University of Pathology (2004). Dyspnea and orthopnea. *Contemporary Medicine*.

[7] Schardt D., Heeg P. (2003). Device for positioning a tumour patient with a tumour in the head or neck region in a heavy ion therapy chamber.

[8] Behr L. V. Patient positioning device.

[9] Wang Z. H., Wang M. An automatic chair for treating head and neck cancer with protons and heavy ions.

[10] Qiu W. Q., Wu Y. S., Lu W., Zhang Z. J., Lan P. Q., Luis A. C. Sitting position fixing device for particle radiotherapy.

[11] Zhang Y. D., Yang Z. K., Guo S. J. Design and kinematic analysis of positioning chair for proton heavy ion radiotherapy.

[12] Zhu F. F., Zhao F. (2018). Research on development and design of family emergency products based on U-TRIZ theory. *Packaging Engineering*.

[13] Zhang W. C., Zhao M., Chen J. (2014). U-TRIZ based SAFC analysis model. *Technology Economics*.

[14] Vincent C. J., Li Y., Blandford A. (2014). Integration of human factors and ergonomics during medical device design and development: it's all about communication. *Applied Ergonomics*.

[15] Shentu J. H. (2008). Medical device design and example application based on ergonomics. *Business: Financial Research*.

[16] Zhao Y. J., Zhang Y. D., Jin J. G. (2009). Method for solving robot workspace based on MATLAB. *Mechanical Science and Technology for Aerospace Engineering*.

[17] Zhang Y. D., Dai X. S., Guo S. J. Preliminary study of the robotic couch for prostate cancer in proton/heavy radiotherapy.

[18] Hsiao S.-w., Chiu F.-y., Chen C. S. (2008). Applying aesthetics measurement to product design. *International Journal of Industrial Ergonomics*.

[19] Sonderegger A., Sauer J. (2010). The influence of design aesthetics in usability testing: effects on user performance and perceived usability. *Applied Ergonomics*.

[20] Chaffin D. B. (2005). Improving digital human modelling for proactive ergonomics in design. *Ergonomics*.

[21] Conn V. S., Hafdahl A. R., Cooper P. S., Brown L. M., Lusk S. L. (2009). Meta-analysis of workplace physical activity interventions. *American Journal of Preventive Medicine*.

